# Benevolent Ideology and Women’s Economic Decision-Making: When Sexism Is Hurting Men’s Wallet

**DOI:** 10.1371/journal.pone.0148629

**Published:** 2016-02-12

**Authors:** Aude Silvestre, Marie Sarlet, Johanne Huart, Benoit Dardenne

**Affiliations:** 1 PsyNCog Research Unit, University of Liege, Liege, Belgium; 2 Fonds de la Recherche Scientifique-FNRS, Brussels, Belgium; Middlesex University London, UNITED KINGDOM

## Abstract

Can ideology, as a widespread “expectation creator,” impact economic decisions? In two studies we investigated the influence of the Benevolent Sexism (BS) ideology (which dictates that men should provide for passive and nurtured women) on women’s economic decision-making. In Study 1, using a Dictator Game in which women decided how to share amounts of money with men, results of a Generalized Linear Mixed Model analysis show that higher endorsement of BS and contextual expectations of benevolence were associated with more very unequal offers. Similarly, in an Ultimatum Game in which women received monetary offers from men, Study 2’s Generalized Linear Mixed Model’s results revealed that BS led women to reject more very unequal offers. If women’s endorsement of BS ideology and expectations of benevolence prove contrary to reality, they may strike back at men. These findings show that BS ideology creates expectations that shape male-female relationships in a way that could be prejudicial to men.

## Introduction

Recent research has shown that expectations and beliefs shape economic decision-making. For instance, in a bargaining task, participants who have an expectation of fairness reject more unfair offers than participants who expect low offers [1,2] and this is important because people in general and women in particular are expected to be generous [3,4]. Similarly, female recipients anticipate less than the amount male participants anticipate [5]. Sexist ideologies are also known to create interpersonal and intergroup expectations [6,7]. The present research aims to show that the Benevolent Sexism (BS) ideology can shape inter-sex women’s economic decision-making.

BS ideology describes women as delicate, as jewels, and as more sophisticated than men. Accordingly, they should be cherished, protected, and taken care of by men. This description and the prescription of niceness to women imply that they are not able to take care of themselves, that is, that they are incompetent. By casting women in a traditional, subordinate and very passive role, despite its subjectively positive tone, BS plays a role in maintaining male-female inequality [8]. Research on the effects of exposure to this ideology shows that it undermines women’s performance [9] by inducing intrusive and disturbing thoughts about performing badly, which, in turn, can alter executive brain responses [10,11]. Moreover, when women do not display the expected, desired and even requested qualities and do not act appropriately, they can suffer hostility [12,13] or a *backlash effect* [14,15].

### Benevolent Sexism as an Attractive Ideology

Because BS ideology is ubiquitous, and despite its deleterious effects on women’s performance, BS behaviors may sometimes be demanded, at least in some contexts, by the women themselves [16,17]. It has been recently demonstrated that both male and female participants who highly endorse BS ideology recommended that men engage in more benevolent and paternalistic behaviors toward women, at least in a romantic context [7,18]. Indeed, such behaviors also have some advantages for women: they could protect them against and are a tool for coping with masculine hostility [19,20]. For instance, [21] demonstrated that women who were threatened by men’s hostility were more likely to approve of BS ideology (see also [22]).

In line with the literature reviewed above, we argue that the endorsement of BS ideology should lead women to expect men to offer them resources, such as money. Consequently, if women hold the purse strings and have to share money with men, endorsement of BS ideology should lead them to make quite unequal or unfair offers to men. Alternatively, if men hold the purse strings and do the sharing, the BS ideology should lead women to reject very unequal (counter-ideological) offers when rejection means that neither receives anything (as the case in some economic games). Research has shown that such rejections are a way of punishing the stingy person [23]. In other words, when their ideology clashes with reality, higher BS women would no longer stay passive but would strike back at men. Just as women who are not warm enough are rejected, men who do not live up to BS expectations and ideology could be turned down in a *backlash effect* too [15].

### Facial Appearance of Benevolence

In addition to the effect of women’s endorsement of BS, inter-sex economic decision-making could be influenced by the predictions women make about men’s own degree of ideological endorsement of BS. Because 50ms of exposure to a face is sufficient to infer traits such as “competence” and “trustworthiness” [24,25], we suspect that men’s facial appearance could signal BS and in turn would create an expectation of protection, sharing, nurturance, and financial care. More specifically, we propose that women should offer less money to “facially BS” men and expect them to be generous when they decide to share money.

## The Present Research

In two experiments using economic games, we tested the hypothesis that BS ideology would affect women’s inter-sex economic decision-making. In Study 1, a standard Dictator Game (DG) required female participants to share a sum of money with (non-anonymous) male recipients. They had to choose between making equal offers (fair), unequal offers (in which they keep more than they give but in quite reasonable proportions, slightly unfair), and very unequal offers (in which they keep by far the biggest share of the pie, highly unfair). According to our hypothesis, the more a female participant endorses BS ideology and the more BS the male recipient’s face appears to be, the more likely she would be to make very unequal offers.

Study 2 staged a standard Ultimatum Game (UG) in which the roles were reversed: female participants had to either accept or reject the division of a sum of money made by (non-anonymous) male proposers. A rejection has the consequence that the money is “lost” for both participants [26]. For very unequal offers, we predicted that higher BS women would be more likely to reject them (because counter-ideological) than less BS women, and that a proposer’s facial appearance of BS should induce the same effect (because counter-expectancy).

### Ethics statement

Both studies were carried out in accordance with the Declaration of Helsinki. The studies received ethical approval from the Ethics Committee of the Department of Psychology and all participants provided written informed consent after the nature of the procedures had been fully explained.

### Study 1

Participants were told that they were about to take part in a study on people’s beliefs about money. They were told that the study consisted in first completing some questionnaires and then role-playing a computer-based bargaining task in which they had to share (hypothetical) money with other people. In order to make the experiment as realistic as possible, we explained that the recipients who would appear on the screen had been photographed earlier by the experimenter and asked the participants to allow us to photograph them for some later studies. To prevent suspicion (because all recipients were male), we told participants that, depending on the experimental condition, recipients would be only male, only female, or mixed. We carefully checked to make sure they understood the structure of the game. For all the participants, they first completed a demographic questionnaire, then they had to complete the French version of the Ambivalent Sexism Inventory (ASI, see [27,28]). At the end of the session, they engaged in a dictator game.

#### Materials and method

Participants were 75 female undergraduates (mean age = 21.12; *SD* = 2.25), all of whom were native French speakers. They were approached by a female experimenter in different places around the university campus.

Prior to the current study, we ran a pretest with judges who were similar to our participants (*N* = 10) on 39 photographs randomly selected after a Google search, all very similar in size and color. Judges had to respond to the statements of the BS subscale of the French version of the ASI ([27–28], see below) by guessing the answer the 39 photographed men would have given to each item on a Likert-like scale ranging from 1 (*the man in the photograph does not at all agree with that statement*) to 9 (*the man in the photograph completely agrees with that statement*). Judges also evaluated each face’s attractiveness and distinctiveness on a Likert-like scale ranging from 1 (*weakly*) to 9 (*strongly*).

Twelve pictures were selected from the picture set to represent the spectrum of benevolence. For these 12 pictures, average men’s perceived benevolence (the mean from the guesses at the BS items) varied from 4.55 to 6.51, with a grand mean of 5.39 (*SD* = .80). Judges’ intraclass correlation was .68, *F*(11, 99) = 3.53, *p* < .001. Attractiveness and distinctiveness being strongly correlated (*r*(12) = .91, *p* < .001), they were then averaged into an attractiveness/distinctiveness index. This index and face’s perceived BS were not correlated, *r*(12) = .34, *p* = .29.

Participants in the study first completed the 22-item French-language version of the ASI [28] composed of two 11-item subscales, one evaluating to what extent an individual endorses hostile sexism and the other evaluating benevolent sexism. For each item, participants had to respond on a Likert-like scale ranging from 0 (*strongly disagree*) to 5 (*strongly agree*). For example, the benevolent sexist subscale contains items such as *“In a disaster*, *women ought to be rescued before men”* and *“Women*, *as compared to men*, *tend to have a more refined sense of culture and good taste*.*”* Examples of hostile sexist items include *“Women are too easily offended”* and *“Most women fail to appreciate fully all that men do for them*.*”* Consistent with prior studies [9], the hostile sexism (*α* = .80) and benevolent sexism (*α* = .81) subscales were positively correlated, *r*(75) = .44, *p* < .001.

Participants then played a standard single-shot DG in the role of the proposer. Participants were seated in front of a computer screen displaying the 12 selected faces and received a sheet of paper presenting 24 possible offers based on a €30 total. Eight offers were fair to the photographed men (they received from 40% to 50% of the €30 total sum while the participant kept the rest); eight offers were quite unequal (the men received from 27% to 33% of the stake); and, finally, eight offers were very unequal (the men received only 18% to 22% of the total stake). The task was to make an offer to each of the face. Each specific offer could be made only once and a specific face could receive only one offer. The dependent variable was the fairness of the offer (equal, unequal, or very unequal), recorded by the computer in a data file [29]. At the end of the experiment, participants were fully debriefed, probed for suspicion, and thanked for their participation.

#### Results

Overall, participants made 33% equal offers, 29% unequal offers, and 38% very unequal offers (S1 File). Because observations are dependent within the same subjects across repeated measures, we conducted a Generalized Linear Mixed Model analysis [30–32] with multinomial distribution (as there are 3 categories of response, coded 1 = equal, 2 = unequal, and 3 = very unequal) and a cumulative logit link function to predict participants’ proposals. The cumulative logit estimates the impact of a predictor on the odds (or probability) of being at or above a category on the (ordinal) outcome variable. Faces’ perceived BS and participants’ endorsement of BS were the independent continuous variables (fixed effects, mean centered). Interaction between these variables was also entered as a predictor. Faces’ attractiveness/distinctiveness and participants’ hostile sexism scores were mean-centered and entered into the analysis as continuous covariates (fixed effect treated as covariates). Intercept was the random effect and we used an unstructured random effect covariance type (each variance and each covariance is estimated uniquely from the data).

**Fig 1:**
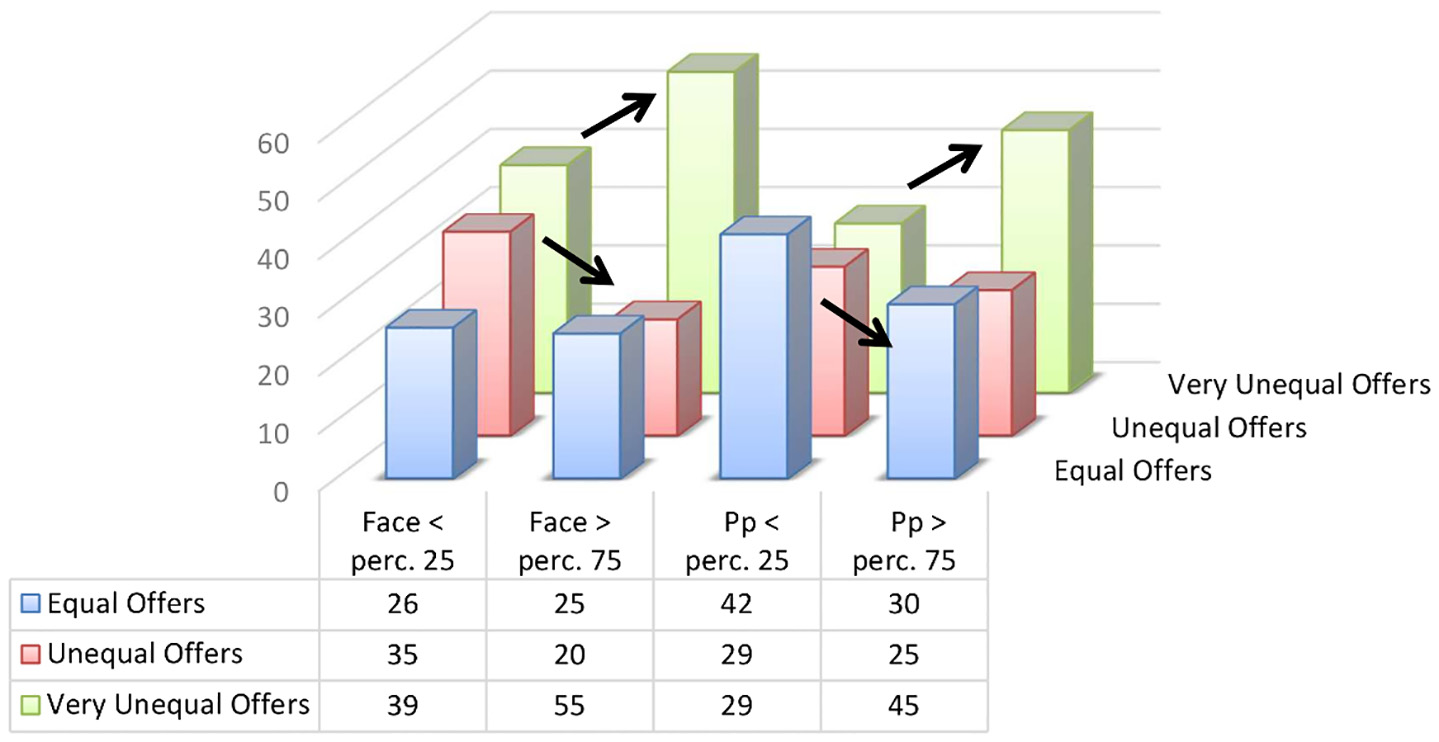
Distribution of offers on the extremes of participants’ and face’s Benevolent Sexism. Percentage of equal, unequal, and very unequal offers made by participants below the 25th percentile and above the 75th percentile in endorsement of benevolence and the same percentages for faces whose perceived sexist benevolence was below the 25th percentile and above the 75th percentile (based on raw data, Study 1).

Tests of model effects revealed the expected effects of both participants’ and faces’ BS. Parameter estimates showed that, holding all the other variables constant, a one-unit increase in participants’ endorsement of BS resulted in 28.5% greater odds of switching from a more to a less equal offer (log-odds = 0.251, SE = 0.080, *t* = 3.116, *p* = .002). Similarly, a one-unit increase in a face’s BS resulted in 41.8% greater odds of switching from a more to a less equal offer (log-odds = 0.349, SE = 0.086, *t* = 4.049, *p* < .001). To better visualize these effects, Fig 1 displays the percentages (raw data) of equal, unequal and very unequal offers made by participants who were below the 25th percentile and above the 75th percentile in BS endorsement and the same percentages for faces whose perceived BS was below the 25th percentile and above the 75th percentile. Cramer’s V = .17 and .18, respectively for BS endorsement and faces’ BS (both *p*s = .001), revealing that the distribution of offers is not the same in the lower and upper percentile distributions. Closer inspection of the distributions (see Fig 1) reveals that for both BS endorsement and faces’ BS, there is a significant increase in the proportion of very unequal offers made between the 25^th^ and the 75^th^ percentile (*p*s < .05). For BS endorsement, there is a significant decrease of equal offers made between the 25^th^ and the 75^th^ percentile, while for faces’ BS, there is a significant decrease of unequal offers made between the 25^th^ and the 75^th^ percentile (*p*s < .05).

The interaction between participants’ and faces’ BS was not significant (*p* = .89). No other effect was significant (*p*s > .079).

#### Discussion

The results show that both the endorsement of BS and faces’ perceived BS had an influence on the offers made in economic decision-making. The higher the endorsement of BS and the higher a face’s subjective BS, the more very unequal offers women made to men: *Men should provide; therefore I take a lot*. Study 2 was designed to examine what happens when men’s and women’s roles are reversed.

### Study 2

The procedure and material were very similar to those used in Study 1. We told participants that they were about to take part in an experiment consisting of a role-play in a simple bargaining computer game in which they had to accept or refuse (hypothetical) monetary offers from other players. After the participants completed a demographic questionnaire, they filled in the French version of the ASI [27] and began the Ultimatum Game (UG). The proposers in the UG were all the 39 photographed men from the pretest (see Study 1), who were presented on a computer screen. Face’s perceived BS, attractiveness as well as distinctiveness were collected at the pretest (see Study 1). The experimenter carefully checked to make sure participants understood the structure of the game.

#### Materials and method

Participants were 48 female undergraduates (mean age = 21.88; *SD* = 2.06), all of whom were native French speakers. They were approached by a female experimenter in different places around the university campus.

Contrary to Study 1 in which only a subset of photographs was presented, the full set of the 39 pre-tested photographs (see Study 1) was presented to participants in Study 2. Across the 39 pictures, the mean perceived BS (the mean from the guesses at the BS items, see Study 1) varied from 4.23 to 6.90, with a grand mean of 5.56 (*SD* = .76). Judges’ intraclass correlation was .66, *F*(38, 351) = 3.82, *p* < .001. Attractiveness and distinctiveness being strongly correlated (*r* = .94, *p* < .001), they were then averaged into an attractiveness/distinctiveness index. This index and a face’s perceived BS were not correlated, *r* = .23, *p* = .17.

Participants played a standard single-shot UG in the role of the responder. We created 39 different offers based on a total sum of €20. As in Study 1, one third of the offers were equal, (moderately) unequal and very unequal. Each participant played 39 trials in which each offer was made by one and only one of the 39 pictures. All dyads of offers and pictures were randomly presented to each participant. First, one of the 39 men’s faces appeared in full-screen mode for 3000ms. On the next screen, a reduced version of the picture appeared, with the offer below it. For example, the man’s face was accompanied by the sentence: “*This player received €20 and decided to split it as follows*: *€3*.*40 for you and €16*.*60 for him*.*”* Then the question *“Do you accept his offer*?*”* appeared. Participants had to respond to the offer by pressing key 1 to accept or key 2 to refuse. If the participant pressed 1, each player received the agreed-upon sum; if she pressed 2, both players received nothing. After each participant’s decision, a screen would display the amount of money she had received for 2000ms. In our example, the display would read *“You received €3*.*40*,*”* or *“You both received €0*.*”* The dependent variable, the number of accepted offers, was recorded by the computer in a data file [29]. At the end of the experiment, participants were fully debriefed, probed for suspicion, and thanked for their participation.

#### Results

Overall, participants accepted 45.9% of the offers. Although 88% of the equal offers were accepted, only 5% of the very unequal offers were accepted, with the (moderately) unequal offers falling in between (45% accepted) (S1 File). As in Study 1, to predict participants’ acceptance of the offers (coded 0 when the offer was denied and 1 when the offer was accepted), we conducted a Generalized Linear Mixed Model analysis with a binomial distribution (as there are 2 categories of response) and a binary logit link. The binary logit estimates the impact of a predictor on the odds (or probability) of switching from one category of response to the other. Fairness of the offer (coded -1 if equal, 0 if moderately unequal, and 1 if very unequal -with this last coding being the reference category), faces’ BS, and participants’ endorsement of BS (fixed effects) were independent variables (mean centered). All interactions between these predictors were then entered into the model. Faces’ attractiveness/distinctiveness and participants’ hostile sexism score were mean-centered and entered into the analysis as continuous covariates (fixed effect treated as covariates). Intercept was the random effect and we used an unstructured random effect covariance type.

Test of the model effects revealed the expected interaction between the fairness of the offer and participants’ BS (Wald = 23.97, *df* = 2, *p* < .001). As can be seen in Table 1, for the very unequal offers, a one-unit increase in participants’ endorsement of BS resulted in 30.4% lower odds of accepting an offer. However, for the unequal and equal offers, the effects of participants’ BS was reverse: A one-unit increase in participants’ endorsement of BS resulted in 69.1% and 88.4% higher odds of accepting an offer, respectively for (moderately) unequal and equal offer. A marginally significant interaction revealed that the same pattern emerged for face’s BS as well (Wald = 2.902, *df* = 2, *p* = .055). For the very unequal offers (see Table 1), a one-unit increase in face’s BS resulted in 22.3% lower odds of accepting an offer. However, for the unequal and equal offer, the effects of face’s BS was again reverse: A one-unit increase in face’s BS resulted in 74.8% and 78.4% higher odds of accepting an offer, respectively for (moderately) unequal and equal offer.

**Table 1:** Parameters estimates for participants’ and face’s Benevolent Sexism, separately for relative fairness of the offers (Study 2).

Predictors	*b* (log-odds)	SE*(b)*	Odds ratio	*T*	*p*
Pp’s BS for very unequal offers	-0.827	0.376	0.437	-2.203	.028
Pp’s BS for unequal offers	0.804	0.379	2.235	2.123	.034
Pp’s BS for equal offers	2.031	0.410	7.621	4.958	.001
Face’s BS for very unequal offers	-1.248	0.483	0.287	-2.585	.010
Face’s BS for unequal offers	1.087	0.493	2.964	2.206	.028
Face’s BS for equal offers	1.291	0.538	3.637	2.402	.016

Pp = Participants; BS = Benevolent Sexism.

Among the covariates, only the faces’ attractiveness/distinctiveness effect was significant (log-odds = -.080, SE = 0.037, *t* = -2.190, *p* = 029). Controlling for all the other predictors, offers made by attractive and distinctive faces were more likely to be rejected than offers from less attractive and distinctive ones, although simple bivariate correlation over all the trials revealed a significant positive association between attractiveness/distinctiveness and acceptance (*r* = .06, *p* = .01). For participants’ hostile sexism, *p* = .69. Not surprisingly, tests of the model effects also revealed a significant effect of the fairness of the offer (Wald = 117.41, *df* = 3, *p* < .001). No other effect was significant (all *p*s > .39)

#### Discussion

When offers were very unequal, both the endorsement and the ‘facial promise’ of BS led participants to reject them. *Man should provide; he did not; I prefer to have nothing but he neither*. In this UG, both the participant and the purported proposer are (hypothetically) “punished” because the participant’s refusal means that both get nothing. UG research has shown that, contrary to the economic rational predictions stating that it is better to have little money than nothing, people would actually rather have no money at all (by rejecting the offer) than being swindled. “The main tendencies observed were that responders are willing to sacrifice substantial amounts to punish a greedy proposer” ([33], p.331). This effect has been replicated in our study and was accentuated by women’s endorsement of BS as well as by facial’s BS appearance.

We did not predict that BS would lead participants to accept equal or (moderately) unequal (30% of the stake) offers more often (see Table 1). Although we understand that reasoning along the lines of *Man should provide; he did (at least reasonably); therefore I accept the offer* could explain acceptance, it is harder to see why a lesser endorsement or weaker ‘facial promise’ of BS should lead someone to refuse an equal offer and prefer to get nothing! Maybe the answer relates to participants’ degree of feminism or independence which, if high, could make any masculine help seem intolerable. We will return to this issue later.

## General Discussion

Although blatantly positive, research has repeatedly shown that BS has negative consequences on women that can contribute to maintain them in a lower status. In two studies, we show that BS can also have negative consequences for men. In Study 1, in which women participants had to share a sum of money with men, greater endorsement of BS ideology as well as greater BS’ appearance were associated with more unequal offers. In Study 2, women participants rejected more very unequal offers if they scored high in the endorsement of BS ideology or if they had great expectations of BS because of the proposer’s face. Presumably, in both studies, women participants appeared to expect BS from men and behaved accordingly.

### Limitations and future research

In Study 2, BS also leads to a greater acceptance of equal and moderately unequal offers. It makes sense that considering men as nurturers should lead women to accept their offers as long as they are not stingy. But why equal offers are refused by less BS participants or from apparently less BS proposers is more puzzling. One possibility is that low BS participants could also be feminists (or more generally, more independent). In this case, as well as when proposers do not look being voluntarily generous towards women (low BS faces), any financial proposal from men could be seen as unacceptable. Indeed, it has been argued that receiving money in male-female relationships can impose the feeling of a debt that is seen as “social” (not merely financial), a kind of debt for which it is almost impossible to determine when it will be discharged [34]. Further research should explore this topic by evaluating UG participants’ feminism via, for instance, the Feminist Identity Development Scale [35] or the Sex Role Attitudinal Inventory [36]. Another possibility would be to ask participants to explain why they rejected an offer.

In both studies, the interaction between participants’ endorsement of BS and faces’ BS did not reach significance. Future work might also explore whether these instantiations of BS operate independently or in combination. For instance, manipulating the context in which the bargaining games take place might play an important role. A romantic or professional context could dramatically impact the bargaining process. Indeed, according to [7,17], a romantic context, compared to a professional one, could lead women to accept or request more benevolence from men, especially if they appear to be benevolent sexists. More work is obviously needed to better understand whether participant’s endorsement of BS and faces’ perceived BS are really instantiations of the same ideology or pertain to different ideologies.

In both study, women decisions do not have real economic consequences. No real money was to be offered or taken. We however doubt that it could have had a strong impact on our results. First, no participant ever expressed concerns about the procedure and all were quite invested into the task. Second, there are generally very little difference in behavior between the stakes and no-stakes conditions in several economic games [37–40], although evidence is mixed and even sometimes contradictory. In any case, replicating our results with monetary incentivized plays would be important. It could also shed some lights on both potential limitations raised above.

## Conclusion

If BS has harmful effects on women, it can also be deleterious to men. Indeed, women’s endorsement or expectations of BS influence their economic decision-making. First, they are less generous towards men with whom they have to share money. Second, if they do not behave in accordance with the ideology, men too can experience a backlash [15]: Rather than being passive and submissive, at least some women fight back. Presumably, their sense of fairness is heightened when they score higher on BS and when the social situation leads them to expect protection and nurturance from men. Benevolent sexism is an ideology that describes how an ideal world should be: Men should protect and provide for women. When the Princess is frustrated, Prince Charming is call to order.

The present studies also complement recent evidence from experimental economics showing that expectations and social information can influence decision-making [41,1,3–5]. Indeed, our results indicate that purported proposers’ and recipients’ apparent BS facial characteristics can affect economic behavior. We also discovered a further moderator that shapes decision-making: ideology. Ideology, whether endorsed or simply expected because of the situation, plays an important role. If reality is in line with it, everything is fine and acceptance will likely follow the offers. However, if reality does not match ideology, then rejection is a way of punishing the counter-ideological partner. We can state that this is true of benevolent sexism, but we would recommend further exploring the applicability of our conclusion to other ideologies as well.

## Supporting Information

S1 File Data package for Study 1. (ZIP)

S2 File Data package for Study 2. (ZIP)
